# Diffuse intrinsic pontine glioma: poised for progress

**DOI:** 10.3389/fonc.2012.00205

**Published:** 2012-12-28

**Authors:** Katherine E. Warren

**Affiliations:** Pediatric Neuro-Oncology Section, Pediatric Oncology Branch, National Cancer Institute, National Institutes of HealthBethesda, MD, USA

**Keywords:** pons, glioma, brainstem, DIPG, diffuse, intrinsic, pediatric, pontine

## Abstract

Diffuse intrinsic pontine gliomas (DIPGs) are amongst the most challenging tumors to treat. Surgery is not an option, the effects of radiation therapy are temporary, and no chemotherapeutic agent has demonstrated significant efficacy. Numerous clinical trials of new agents and novel therapeutic approaches have been performed over the course of several decades in efforts to improve the outcome of children with DIPG, yet without success. The diagnosis of DIPG is based on radiographic findings in the setting of a typical clinical presentation, and tissue is not routinely obtained as the standard of care. The paradigm for treating children with these tumors has been based on that for supratentorial high-grade gliomas in adults as the biology of these lesions were presumed to be similar. However, recent pivotal studies demonstrate that DIPGs appear to be their own entity. Simply identifying this fact releases a number of constraints and opens opportunities for biologic investigation of these lesions, setting the stage to move forward in identifying DIPG-specific treatments. This review will summarize the current state of knowledge of DIPG, discuss obstacles to therapy, and summarize results of recent biologic studies.

## INTRODUCTION

More than 70% of children with tumors of the central nervous system (CNS) will survive at least 5 years from diagnosis (Smith et al., 2010; Howlader et al., 2011). However, pediatric CNS tumors represent a heterogeneous group of diseases and the dismal survival of select tumor subtypes, such as diffuse intrinsic pontine gliomas (DIPG), is not reflected in this number. The median survival for children with DIPG is less than 1 year from diagnosis (Mandell et al., 1999; Cohen et al., 2011), and no improvement in survival has been realized in more than three decades. The reason for this stagnancy has, at least in part, been attributed to our lack of understanding of the biology of this disease. In the past few years, considerable coordinated and collaborative efforts have been made to address this. Notably, more has been published on the biology and pathophysiology of DIPG in the past 5 years than in all prior years combined, generating a groundswell of excitement and cautious enthusiasm. How to best apply this data to the treatment of children with DIPG remains to be seen, but improved outcome for these patients is anticipated as we move beyond empiric therapy and attempt to bridge the gap between bench and bedside. This review will discuss the current status and our present understanding of this disease.

**FIGURE 1:**
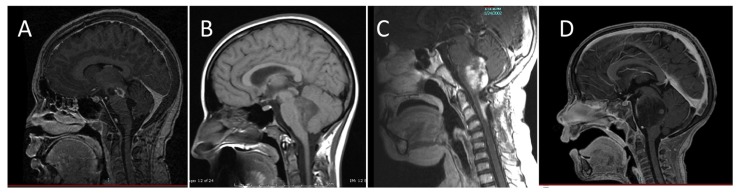
**Classification of brainstem gliomas by MRI appearance**. **(A)** Focal brainstem lesion on T1-weighted post-contrast sagittal image. **(B)** Dorsal exophytic brainstem lesion on sagittal non-contrast MRI. **(C)** Cervicomedullary lesion on T1-weighted post-contrast sagittal image. **(D)** Diffuse intrinsic pontine glioma lesion on T1-weighted post-contrast sagittal image.

Diffuse intrinsic pontine gliomas are the most common brainstem tumors in children, representing 75–80% of pediatric brainstem tumors, and affecting an estimated 200–300 children in the United States each year. While brainstem tumors have sometimes been grouped together as a single entity, magnetic resonance imaging (MRI) has allowed classification of these tumors into distinct subsets of focal, dorsally exophytic, cervicomedullary, or diffusely infiltrating tumors based on imaging characteristics (Epstein and Farmer, 1993; **Figure 1**). The prognosis for children with diffusely infiltrating pontine gliomas is significantly worse than that of other brainstem tumors. The pons contains cranial nerve nuclei and nuclei critical for life-sustaining function, so damage by the tumor or its treatment has tremendous repercussions. Resection is not an option and the tumors are resistant to other therapeutic measures.

## PRESENTATION AND DIAGNOSIS

Diffuse intrinsic pontine glioma is generally a disease of middle childhood, with the majority of children diagnosed between 5 and 10 years of age. Most present with some evidence of brainstem dysfunction or cerebrospinal fluid (CSF) obstruction, although a handful of tumors are identified as incidental findings. Typically, symptoms are present for a short period of time (i.e., <1 month), but it is not unusual to have generalized or subtle symptoms present for several months prior to diagnosis. Commonly reported symptoms include abnormal or limited eye movements, diplopia, an asymmetric smile, clumsiness, difficulty walking, loss of balance, and weakness. Classic findings on clinical examination include the triad of multiple cranial neuropathies, long tract signs (hyperreflexia, clonus, increased tone, presence of a Babinski reflex), and ataxia. Signs and symptoms of increased intracranial pressure may be present due to obstructive hydrocephalus resulting from expansion of the pons. Various other symptoms may occur, including behavioral changes, night terrors, and school difficulties.

**FIGURE 2:**
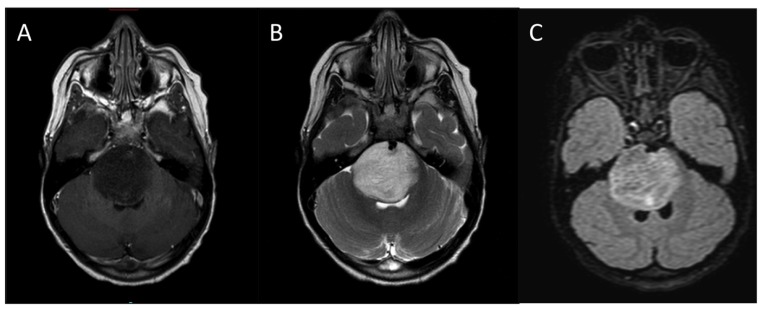
**Typical MRI appearance of DIPG**. **(A)** T1-weighted post contrast, **(B)** T2-weighted, **(C)** FLAIR.

The diagnosis of DIPG is based on characteristic MRI findings in the face of this typical clinical presentation (**Figure 2**). On MRI, the tumor appears as a large expansile brainstem mass as opposed to an extrinsic mass compressing the pons. While there may be an exophytic component due to expansion of the tumor via the path of least resistance, the epicenter of DIPG lies within the pons, and the lesion involves the majority of the pons. DIPGs are hypo- or iso-intense on T1- weighted imaging, hyperintense on T2-weighted imaging, and frequently appear relatively homogeneous on fluid attenuated inversion recovery (FLAIR) sequences. Pinpoint intratumoral hemorrhages are not uncommon. Other MRI features of typical DIPG include ventral involvement of the pons, and encasement of the basilar artery. Contrast-enhancement is variable, but these lesions frequently do not significantly enhance at diagnosis.

Prior to the routine use of MRI, it is estimated that up to 15% of patients diagnosed with DIPG actually had a non-tumor process or non-glial tumor (Jenkin et al., 1987; Epstein and Wisoff, 1988), and biopsy procedures were frequently undertaken for histological confirmation. Although MRI is not 100% specific, the vast majority of children diagnosed with DIPG by MRI do have a diffuse infiltrative glioma. Consequently, in the early 1990s when MRI became widely available, it was proposed that obtaining tissue for histologic confirmation was not necessary in children with a typical clinical presentation and distinctive radiographic findings on MRI (Albright et al., 1993; Cartmill and Punt, 1999). This recommendation was rapidly incorporated as standard practice given the perceived surgical risk in this delicate area, particularly for children with concurrent increased intracranial pressure and those considered poor surgical candidates. Since the available therapies at the time were primarily non-specific cytotoxic agents, the initial repercussions of diagnosis without tissue appeared to be of little consequence.

## HISTOLOGY

**FIGURE 3:**
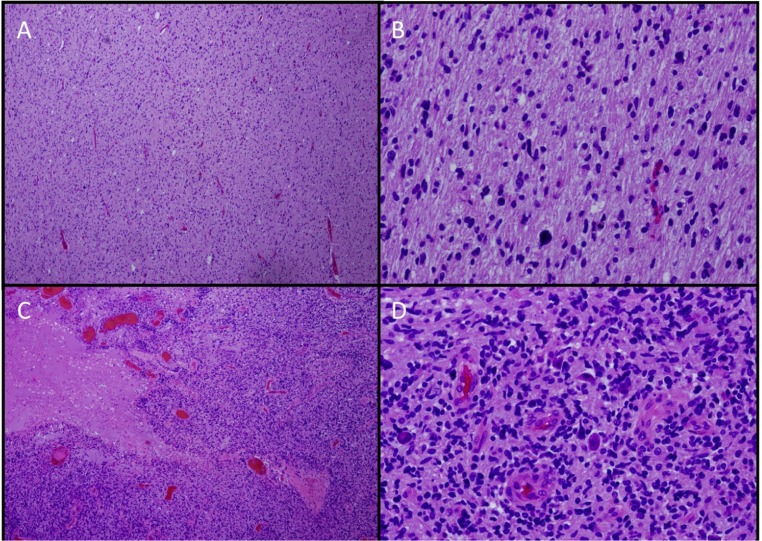
**Histologic geographic variability of DIPG**. ×4 **(A,C)** and ×20 **(B,D)**. Hematoxylin and eosin stains from different sections of a single tumor showing low-grade **(A,B)** and high-grade **(C,D)** areas.

Because of limited tissue availability, our knowledge of DIPG comes primarily from evaluation of autopsy specimens, small biopsy samples obtained from patients with atypical radiographic findings, and biopsy samples obtained from a small number of institutions such as the Institut Gustave-Roussy where biopsy has been routinely performed on children with suspected DIPG since 2003 (Roujeau et al., 2007). The majority of diffusely infiltrating brainstem tumors are fibrillary astrocytomas, histologically resembling malignant gliomas in other locations. Anaplasia, increased mitotic activity, tumor necrosis, and vascular proliferation are often, but not always, present. DIPGs may therefore be classified histologically as fibrillary astrocytomas, World Health Organization (WHO) Grades II–IV (Schumacher et al., 2007), although the utility of grading has been questioned. When biopsies are indicated, they are generally obtained from the most accessible area and may not be representative of the entire tumor as significant geographic variability of these tumors has been reported (Warren et al., 2011c; **Figure 3**). In addition, prognosis is not associated with histological grade (Edwards et al., 1989; Grigsby et al., 1989; Hargrave et al., 2006; Laigle-Donadey et al., 2008).

Tumor cells often appear relatively small, with prominent cytoplasmic intermediate filaments and cell processes, similar to fibrillary astrocytomas in other locations of the brain (Maria et al., 1993). In DIPG, tumor cells tend to pervade normal cells, diffusely expanding the pons and distorting, displacing and destroying nerve fiber tracts that normally course through it (Maria et al., 1993). The tumors tend to spread contiguously, extending to involve the midbrain, medulla, and cerebellar peduncles (Mantravadi et al., 1982; Grigsby et al., 1989). Despite this tendency for local spread, CNS metastasis at diagnosis is not uncommon, with up to 20% of patients reported to have leptomeningeal disease at diagnosis (Donahue et al., 1998; Sethi et al., 2011). This may be an underestimate, as spinal disease is not always investigated in asymptomatic patients. Significantly higher numbers of patients (up to 56%) have evidence of spinal metastases or leptomeningeal dissemination at the time of recurrence or autopsy (Donahue et al., 1998; Gururangan et al., 2006; Sethi et al., 2011).

## STANDARD THERAPY AND DISEASE COURSE

The standard of care for children with newly diagnosed DIPG is focal radiation therapy, using a 1 cm margin to cover microscopic disease, to a total dose of 54–60 Gy administered over 6 weeks, usually in daily (Monday–Friday) 180–200 cGy fractions. Glucocorticoids are frequently administered in an effort to reduce and control edema associated with the tumor and radiation treatment. About 75% of patients will have some improvement in neurological symptoms in response to radiation therapy and steroids, but many patients suffer concomitant adverse effects primarily attributed to steroids. Radiation therapy appears to control tumor growth for a short period of time, prolonging survival by a mean of ~3 months (Haas-Kogan et al., 2011). Patients receiving doses under 50 Gy have a worse outcome compared to those receiving higher doses (Lee, 1975; Kim et al., 1980; Litman et al., 1980). Total radiation doses higher than 60 Gy have been evaluated. Initial studies utilizing hyperfractionated radiation therapy with total doses up to 72 Gy suggested a modest improvement in survival of children with brainstem gliomas compared to radiation alone, and compared to radiation with neoadjuvant or adjuvant chemotherapy (Edwards et al., 1989; Freeman et al., 1993; Packer et al., 1993). However, subsequent trials using radiation doses up to 78 Gy did not confirm this finding (Freeman et al., 1993; Packer et al., 1994; Mandell et al., 1999). While radiation therapy appears to offer some benefit to children with DIPG, no radiosensitizing agent has improved outcome (Mandell et al., 1999; Marcus et al., 2003; Sanghavi et al., 2003; Bernier-Chastagner et al., 2005).

Within 3–8 months after completion of radiation therapy, most children with DIPG will have clinical or radiographic evidence of disease progression. The pattern of failure is generally local. In one study, 25% of cases with disease progression involved the irradiated volume, while 75% involved the margin of the radiation field (Grigsby et al., 1989). Recently, concern has been raised regarding a suspected increased incidence of distant disease at recurrence with the use of antiangiogenic therapies (Rubenstein et al., 2000; Zuniga et al., 2009), but this has not been clearly demonstrated for patients with DIPG. Additional therapies for DIPG are generally not effective and neurologic deterioration occurs unabated over the ensuing weeks to months.

## CHEMOTHERAPY

Because of inevitable disease progression in the vast majority of children with DIPG, many receive adjuvant chemotherapy, frequently as part of a clinical trial, at some point during their disease course in an attempt to improve survival. However, no chemotherapeutic agent has ever demonstrated a significant improvement in outcome beyond that achieved with standard radiation therapy alone. An early prospective, randomized trial performed 25 years ago compared radiation only versus radiation plus post-radiation chemotherapy using prednisone, CCNU, and vincristine (Jenkin et al., 1987). This study was performed prior to the routine use of MRI and included children with brainstem tumors involving the pons or medulla. Five-year survival was not statistically different between the two arms, with 17% 5-year survival noted in the radiation only arm versus 20% in the radiation plus chemotherapy arm. Of note, these survival rates are higher than those reported in more recent trials; this is likely related to the inclusion of non-DIPG patients. DIPG has been studied in a large number of clinical trials including those evaluating cytotoxic agents, high-dose chemotherapy with stem cell rescue (Dunkel et al., 1998; Bouffet et al., 2000), neoadjuvant chemotherapy (Doz et al., 2002), biologic response modifiers (Warren et al., 2006, 2011a,b) and radiation sensitizers, none of which demonstrated significantly improved outcome. Contemporary studies limiting enrollment to patients with DIPG generally report progression-free survival (PFS) of 5–8 months and 2-year overall survival rates of <10% (Pollack et al., 2007, 2011; Gururangan et al., 2010; Haas-Kogan et al., 2011; **Table 1**).

**Table 1:** Results from recent phase II or non-dose-escalating trials (e.g., pilot) for DIPG.

Reference	Year	Treatment	No. of evaluable patients	Median PFS, EFS, or TTP (month)	Median OS (month)	Median 1-year OS (%)	Comment
Warren et al. (2011a)	2012	XRT, PEG-Intron	32	7.8	11.5	46 ± 9	
Chassot et al. (2012)	2012	TMZ + XRT	21	7.5	11.7	50	Biopsy-proven
Cohen et al. (2011)	2011	TMZ + XRT	58	6.1	9.6	40 ± 6.5	
Haas-Kogan et al. (2011)	2011	Tipifarnib + XRT	40	5.9	8.9	35 ± 7.5	
Pollack et al. (2011)	2011	Gefitinib + XRT	43	7.4		56 ± 7.6	
Sharp et al. (2010)	2010	Metronomic TMZ + XRT	15	5.13	9.8	20 ± 10.3	
Kim et al. (2010)	2010	TMZ + thal + XRT	12	7.2	12.7	58.3	
Jalali et al. (2010)	2010	TMZ + XRT	20	6.9	9.15		
Frappaz et al. (2008)	2008	Pre-XRT chemo (BCNU/MTX)			17		11 patients with symptoms >1 month prior to dx
Sirachainan et al. (2008)	2008	TMZ + XRT, then TMZ + cRA	12	10.2 ± 3	13.5 ± 3. 6	58 ± 14.2	7 patients <5 years; OS 16.2 ± 0.7 months
Korones et al. (2008)	2007	VCR, VP-16, XRT	30		9	27.7 ± 7	
Turner et al. (2007)	2007	Thal + XRT	12	5	9		
Aquino-Parsons et al. (2008)	2006	Carbogen + XRT	7	8	9.6		
Bernier-Chastagner et al. (2005)	2005	Topotecan + XRT	32		8.3	25.5 ± 8	

*DIPG, diffuse intrinsic pontine glioma; DIBG, diffuse intrinsic brainstem glioma; XRT, radiation therapy; PFS, progression-free survival; TMZ, temozolomide; TTP, time to progression; EFS, event-free survival; OS, overall survival; cRA, cis-retinoic acid.*

The majority of recent trials are early (i.e., phase I) clinical trials or single-armed phase II studies that rely on historical controls for comparison (Broniscer et al., 2010; Michalski et al., 2010; Cohen et al., 2011; Geoerger et al., 2011; Haas-Kogan et al., 2011; Warren et al., 2011a). Although most, if not all, studies demonstrate the all-too-recognizable ski-slope Kaplan–Meyer survival curve, a true historical cohort has not been defined. Those studies that include younger children, children with a long history of symptoms prior to diagnosis, and children with neurofibromatosis type 1 (NF-1) may favorably bias results, as will those excluding patients with herniation, disseminated disease, and intratumoral hemorrhage. The eligibility criteria for previous and ongoing studies differ, the definition of typical and atypical DIPG is not standardized, and the definition of response or disease progression varies between, and within, pediatric consortia. There is frequently mismatch between clinical and radiographic findings for an individual patient; while some adhere to radiographic definition of response and progression, others adjust treatment based on clinical findings alone (Hayward et al., 2008). Performing DIPG-specific randomized phase II clinical trials that have adequate power to detect modest improvements (e.g., from 10 to 12 months) in outcome is generally precluded by the relatively small number of patients and the time necessary to reach objective endpoints. Until a more contemporary historical cohort is defined, studies that attempt to identify a modest improvement in survival would therefore require a large number of patients or a randomized control arm involving DIPG patients who receive radiation therapy only, something that is not likely to appeal to patients, families or treating physicians.

## OBSTACLES TO CHEMOTHERAPY

A number of obstacles that may contribute to the lack of efficacy of chemotherapeutic agents are known or suspected. For a drug to be effective against a tumor, you need to have an active agent, delivered to its site of activity, and present in its active form in effective concentrations for a long enough period of time. The cells need to be sensitive to the agent, and if it is a molecularly targeted agent, the target must be present. While this may be stating the obvious, from a practical standpoint, much of this information is unknown when treating a patient with DIPG.

There are several factors that can affect drug levels at the brain tumor site. These include the concentration of drug in the bloodstream, the amount of protein and tissue binding (i.e., it is the free or unbound drug that is active), and the degree of CNS penetration (i.e., how much drug crosses the blood:brain or blood:tumor barriers and diffuses across the brain parenchyma to its site of action). The blood:brain barrier (BBB), a layer of specialized endothelial cells comprising the wall of CNS capillaries, re-enforced by its surrounding basal lamina, pericytes, astrocytes, and microglia (Wolburg et al., 2009), serves to protect the CNS from toxins, limits the CNS penetration of large, hydrophilic substances, and thereby hinders delivery of many chemotherapeutic agents to the tumor site. The blood:tumor barrier may be less restrictive due to disrupted and leaky tumor vessels, but even if agents are able to cross, diffusion of agents across the parenchyma remains limited to only a few millimeters (Morrison and Dedrick, 1986).

Given the lack of adequate tumor-bearing animal models and the ethical constraints of sampling brain tumor tissue in children, the degree of drug penetration at the tumor site and therefore adequate drug dosing remains unknown. With empiric cytotoxic agents, the paradigm is to treat with the maximum tolerated dose (MTD), but this may not result in adequate exposure at the tumor site, and is not applicable to molecularly targeted agents for which an MTD may not be identified.

Several strategies have been employed to overcome the BBB and improve drug delivery to the tumor site. These include the use of high dose chemotherapy with stem cell rescue (Finlay and Zacharoulis, 2005), biologic or osmotic BBB disruption (Hall et al., 2006; Warren et al., 2006), and p-glycoprotein inhibition (Greenberg et al., 2005). Most recently, convection-enhanced delivery has been used to deliver agents directly into the tumor of patients with DIPG (Lonser et al., 2007). Using this technique, agents are delivered under continuous low-pressure via a catheter placed directly in the tumor or tumor bed, and clinical trials in children with DIPG are ongoing.

When treating with chemotherapy, it would be advantageous to assess drug activity or inactivity early in the treatment course so treatment options can be reassessed and patients will not be exposed to additional cycles of inactive agents. Determining the acute effects of an agent, particularly a cytostatic agent, and early efficacy on the tumor and its microenvironment can be difficult. In neuro-oncology, activity of an antitumor agent is frequently assessed using MacDonald criteria (MacDonald et al., 1990) or a variant, with decreased tumor size, decreased steroid use, and improved neurologic symptoms indicative of response. However, there are a number of issues with applying these response criteria to patients with DIPG. These criteria were developed primarily for enhancing supratentorial gliomas, and DIPG frequently do not enhance or they may exhibit a heterogeneous pattern of enhancement. Given their invasive nature and indistinct borders, there is significant interobserver variability using standard tumor measurement criteria when measuring DIPG on MRI (Hayward et al., 2008). Even if the tumor size is reduced by a chemotherapeutic regimen, this is generally not sustained and does not translate into improved survival. Standard MRI cannot reliably differentiate tumor and treatment effects, and phenomena such as pseudoprogression and pseudoresponse complicate interpretation of MR images. For children with DIPG, new enhancement months after radiation therapy may represent treatment effect (e.g., radiation necrosis), tumor progression, or both. These cannot be easily distinguished using standard MRI, resulting in a frustrating predicament for both families and caregivers.

Non-invasive evaluation to identify response to antitumor agents continues to be investigated, and some imaging techniques, such as MR perfusion and magnetic resonance spectroscopy (MRS), have shown promise as predictive or surrogate markers of response in this population (Hipp et al., 2011; Steffen-Smith et al., 2011). In one study, evaluating spectroscopy in 38 children with DIPG, the choline:*N*-acetyl aspartate ratio (CHO:NAA) was shown to be prognostic, with those patients having CHO:NAA higher than the median of 2.1 demonstrating a greater risk of mortality compared to patients with CHO:NAA ≤2.1 (Steffen-Smith et al., 2011). This study also showed that changes in CHO:NAA over time were associated with outcome. Any increase in CHO:NAA was inversely associated with survival (*p* = 0.009), while decreasing CHO:NAA was associated with a decreased risk of death. The greater the change, the more significant the observed effect.

Likewise, perfusion studies were evaluated in a group of DIPG patients. In general, tumor growth is associated with increased vessel density and increased vessel permeability, features that can be evaluated on newer MRI sequences. Increased blood flow to a region of interest may be indicative of increased vascular growth, associated with tumor grade, or associated with malignant transformation. In a recent study by Price et al. (2011) relative cerebral blood volume (rCBV) determined on perfusion imaging correlated with cell proliferation in adults with high-grade gliomas. In a study evaluating perfusion imaging in 34 children with DIPG, increased perfusion at *any* single time point was associated with shorter survival (RR = 4.91; Hipp et al., 2011). In addition, increasing perfusion over time was a poor prognostic factor. Additional imaging techniques such as diffusion tensor imaging are being evaluated in children with DIPG although their clinical utility remains to be seen.

## LACK OF TISSUE

Because of limited tissue availability, very little information on the biology and pathophysiology of DIPG has been available in the literature (Louis et al., 1993; Cheng et al., 1999; Gilbertson et al., 2003) until recently. The importance of understanding the biology of DIPG has been brought to the forefront with the development of molecularly targeted agents. No molecularly targeted agent has been shown to significantly improve survival in a clinical trial for children with DIPG. This includes therapeutic agents aimed at targets defined in adult high-grade gliomas, including platelet-derived growth factor receptor (PDGFR; Pollack et al., 2007), epidermal growth factor receptor (EGFR; Geoerger et al., 2011; Pollack et al., 2011), vascular endothelial growth factor receptor-2 (VEGFR2) (Broniscer et al., 2010), and farnesyl transferase (Haas-Kogan et al., 2011). Determining why these agents fail is key to advancing the use of molecularly targeted agents in general for the treatment of children with DIPG.

An unprecedented number of studies on the biology of DIPG have been published in the past few years (Joshi et al., 2008; Paugh et al., 2010, 2011; Zarghooni et al., 2010; Barrow et al., 2011; Monje et al., 2011; Warren et al., 2011c). These studies give insight as to the possible cell of origin, genetic profiling, driver mutations, and oncogenic alterations. While the etiology and exact pathophysiology of DIPG remain to be determined, critical pathways and potential treatment targets have been identified, and critical conclusions can be drawn: (1) pediatric DIPGs differ from adult high-grade gliomas, (2) pediatric DIPGs differ from pediatric supratentorial high-grade gliomas, (3) genomic studies of DIPG demonstrate aberrations in druggable targets, (4) significant interpatient and intrapatient variability exists, and (5) the tumor microenvironment appears to play a key role in DIPG tumorigenesis.

An intriguing characteristic of DIPG is the predominant age group affected, with a peak incidence in middle childhood, suggesting an etiology associated with development. In efforts to define a potential cell of origin, Monje et al. (2011) examined the spatial and temporal distribution of neural precursor cells in the human brainstem. They described a distinct cell population in the ventral pons that is Nestin and Vimentin immunopositive (both markers of primitive neuroectodermal cell types in the developing and post-natal CNS); approximately half of these cells are also positive for the basic helix-loop-helix transcription factor Olig2 (classically associated with oligodendroglial lineage precursor cells). They demonstrated that the density of this Nestin+ Vimentin+ Olig2+ cell type normally changes during childhood. It is present in all ventral brainstem structures during infancy, decreases by 2 years of age, and then increases again during middle childhood. What regulates the changing density of this cell population in humans is unknown. However, using a mouse model, Monje et al. (2011) determined that Hedgehog signaling drives proliferation of Olig2+ cells in the ventral pons of mice. In addition, Hedgehog activity and Hedgehog-responsive cells increased in the ventral pons of the mouse during the time period corresponding to middle childhood in humans. These studies suggest that the nature of neural precursor cells in the ventral pons and the microenvironment within the developing brain may influence the disease process.

Puget et al. (2012) also implicated the Sonic Hedgehog pathway in a trial in which they performed genomic studies on a large number (*n* = 61) of newly diagnosed children with DIPG. In this study, DIPG was distinguished from high-grade gliomas by several genes involved in the Sonic Hedgehog pathway. The authors suggest that the gene expression signatures of DIPG were associated with “differential reprogramming of embryonic signaling organizers.” They demonstrated involvement of two distinct oncogenic pathways that resulted in two biological DIPG subgroups, including one group with an oligodendroglial phenotype that was associated with PDGFRA gain or amplification, and another group referred to as the mesenchymal and pro-angiogenic phenotype that was associated with higher expression of STAT3.

Both the Monje and Puget studies implicate altered gene expression during development as potentially key steps in DIPG pathogenesis. Histones play an important role in gene regulation, influencing chromatin structure and accessibility, and post-translational modifications of the histone tail play a role in epigenetic regulation of gene expression. Notably, two recent studies demonstrated somatic mutations in histone H3.3 associated with DIPG (Khuong-Quang et al., 2012; Wu et al., 2012). Wu et al. (2012) performed whole genome sequencing on DNA of seven patients with DIPG and showed that four of these seven had a mutation in *H3F3A* (the gene that encodes the H3.3 protein) or *HIST1H3B* (gene which encodes H3.1) that resulted in a K27M substitution (lysine replaced by methionine at amino acid 27). They expanded this study in 43 additional DIPG patients and found that 78% of DIPG patients demonstrated K27M substitutions in *H3F3A* or *HIST1H3B* compared to only 22% of non-brainstem glioma patients. Similarly, Khuong-Quang et al. (2012) demonstrated that 71% of 42 DIPG patients had the K27M mutation compared to 14% of supratentorial glioblastomas. They also noted that patients with wild type H3.3 had better overall survival. Interestingly, H3.3 is located on Chromosome 1q, an area commonly gained in DIPG (Barrow et al., 2011; Warren et al., 2011c). However, in the study by Wu et al. (2012), there was no significant correlation between the presence of *H3F3A* mutations and gain of chromosome 1q. Lysine 27 of the histone H3 tail is also a key residue for epigenetic regulation of neural precursor cell differentiation (Liu and Casaccia, 2010).

Several genomic studies have identified a number of chromosomal aberrations and targets in DIPG, including PDGFRA, MDM4, MYCN, EGFR, MET, KRAS, CDK4, amongst others (Paugh et al., 2010, 2011; Zarghooni et al., 2010; Barrow et al., 2011; Warren et al., 2011c; Grill et al., 2012; Li et al., 2012). Not surprisingly, many of the identified aberrations involve genes that regulate cell growth, cell death, and repair pathways. Rather than describe each of these in detail, it is important to realize that, although these studies significantly contribute to our knowledge and understanding of the biology of DIPG, the number of samples is relatively small. The glaring fact is that no one target is identified in all tumor cells of all patients with DIPG. Treating a single target will therefore unlikely result in a significantly improved outcome for these patients. Indeed, clinical trials evaluating individual molecularly targeted agents have been performed in children with DIPG without success. Precise reasons for the lack of efficacy are unknown, but in most, if not all, studies, it is unknown if the target was expressed, was insufficiently expressed, or whether drug exposure at the target site was adequate. Presumably, combinations of several targeted agents will be necessary to observe an effect given the multiple chromosomal alterations found in individual patient samples.

## BIOPSY

In contrast to the majority of centers in the United States, routine biopsy of children with suspected DIPG has been performed in Europe since 2003 (Roujeau et al., 2007). In the initial report detailing their experience in 24 children, the investigators report morbidity in 2 children (cranial nerve palsy, worsening hemiparesis) which was reversible, and no incidents of mortality, concluding that the procedure was relatively safe in experienced hands using modern neurosurgical technique (Roujeau et al., 2007). Given this demonstration of relative safety, along with the significant information obtained, the ability to perform genomic testing on small tissue samples, the identification of potentially druggable targets and known interpatient heterogeneity, there is a movement within the pediatric neuro-oncology community to push for routine biopsy of patients with suspected DIPG (MacDonald, 2012; Rutka, 2012) although this remains under debate (Boop, 2011).

Because the vast majority of samples biopsied are malignant gliomas, the primary purpose of the biopsy in patients with a typical clinical presentation and typical radiographic appearance would not necessarily be for histologic confirmation, although this would be important for those with atypical features, particularly since brainstem PNET can mimic DIPG radiographically (Sufit et al., 2012). Rather, the major question to be addressed is whether or not treatment chosen based on biopsy results can improve the outcome of these children. Intrapatient heterogeneity of DIPG has been demonstrated, and defining where to biopsy and how representative these results are of the tumor need to be broached. At the very least, routine biopsy would supply additional tissue for study, may enhance our understanding of the disease, and enrich the datasets gleaned from clinical trials.

## CONCLUSION

An unprecedented number of biologic and genomic studies, generating considerable novel and exciting data, have been performed on DIPG over the past few years, primarily due to a number of collaborative efforts. We now know that DIPGs are a distinct entity, biologically different from both adult high-grade gliomas and pediatric supratentorial high-grade gliomas. We know that genomic mutations occur in DIPG, resulting in a number of druggable targets. However, we also know that no single target defines DIPG, and significant inter- and intrapatient variability exists. Our challenge now is to select appropriate targets, treat with agents at doses that will result in adequate exposure at the site of action, and rapidly identify drug efficacy or lack of response in individual patients. Until we are able to non-invasively identify targets, obtaining tissue from patients will be important for selecting appropriate agents, so that children with DIPG will only be exposed to those agents that have any chance of benefit. Tumor assessment, identification of tumor targets, selection of appropriate agents, and determination of adequate dosing should inform treatment selection, and pre-treatment determination of this data may become the new study paradigm for the next generation of DIPG clinical trials.

It is imperative that we continue to embrace global collaborations given the relatively small numbers of patients. It is key to be able to perform trials and identify efficacious treatment paradigms quickly. We need to be able to compare results from different clinical trials; to do this, similar eligibility and response criteria are necessary, and it is important to identify an appropriate historical cohort. Finally, it is necessary to change our mindset, and not be mired in historical outcomes for children with DIPG, as recent data opens a host of avenues for promising approaches.

## Conflict of Interest Statement

The author declares that the research was conducted in the absence of any commercial or financial relationships that could be construed as a potential conflict of interest.
